# A boy with homozygous microdeletion of *NEUROG1* presents with a congenital cranial dysinnervation disorder [Moebius syndrome variant]

**DOI:** 10.1186/1744-9081-9-7

**Published:** 2013-02-18

**Authors:** Julia C Schröder, Anne K Läßig, Danuta Galetzka, Angelika Peters, John C Castle, Stefan Diederich, Ulrich Zechner, Wibke Müller-Forell, Annerose Keilmann, Oliver Bartsch

**Affiliations:** 1 Institute of Human Genetics, University Medical Centre of the Johannes Gutenberg University Mainz, Langenbeckstrasse 1, D-55101, Mainz, Germany; 2 Division of Communication Disorders, University Medical Centre of the Johannes Gutenberg University Mainz, Mainz, Germany; 3 TRON-Translational Oncology at the University Medical Centre of the Johannes Gutenberg University Mainz, Mainz, Germany; 4 Institute of Neuroradiology, University Medical Centre of the Johannes Gutenberg University Mainz, Mainz, Germany

**Keywords:** Congenital cranial dysinnervation disorder, Moebius syndrome variant, Mondini dysplasia, Sensorineural deafness, Disorder of oral motor function, Aplasia/hypoplasia of cranial sensory ganglia, Aplasia/hypoplasia of cranial nerves V and VIII, *NEUROG1*, Homozygous deletion, Autosomal recessive

## Abstract

**Background:**

We report on a 6-year-old Turkish boy with profound sensorineural deafness, balance disorder, severe disorder of oral motor function, and mild developmental delay. Further findings included scaphocephaly, plagiocephaly, long palpebral fissures, high narrow palate, low-set posteriorly rotated ears, torticollis, hypoplastic genitalia and faulty foot posture. Parents were consanguineous.

**Methods and results:**

Computed tomography and magnetic resonance imaging showed bilateral single widened cochlear turn, narrowing of the internal auditory canal, and bilateral truncation of the vestibulo-cochlear nerve. Microarray analysis and next generation sequencing showed a homozygous deletion of chromosome 5q31.1 spanning 115.3 kb and including three genes: *NEUROG1* (encoding neurogenin 1), *DCNP1* (dendritic cell nuclear protein 1, *C5ORF20*) and *TIFAB* (TIFA-related protein). The inability to chew and swallow, deafness and balance disorder represented congenital palsies of cranial nerves V (trigeminal nerve) and VIII (vestibulo-cochlear nerve) and thus a congenital cranial dysinnervation disorder.

**Conclusions:**

Based on reported phenotypes of *neurog1* null mutant mice and other vertebrates, we strongly propose *NEUROG1* as the causative gene in this boy. The human *NEUROG1* resides within the DFNB60 locus for non-syndromic autosomal recessive deafness on chromosome 5q22-q31, but linkage data have excluded it from being causative in the DFNB60 patients. Given its large size (35 Mb, >100 genes), the 5q22-q31 area could harbor more than one deafness gene. We propose NEUROG1 as a new gene for syndromic autosomal recessive hearing loss and congenital cranial dysinnervation disorder including cranial nerves V and VIII.

## Background

The term congenital cranial dysinnervation disorder (CCDD) describes a heterogeneous group of neurodevelopmental diseases affecting the cranial nerves and its nuclei [1]. At present, a total of 10 phenotypes fall under the CCDD umbrella, including congenital ptosis, Duane syndrome, horizontal gaze palsy, and congenital facial palsy [2]. Some authors also include Moebius syndrome, which is defined by affection of cranial nerves VI and VII, and the Moebius syndrome variants (predominant affection of cranial nerves other than VI and VII) in the entity of CCDD [1,2]. In CCDD, seven disease genes have been identified to date, but a number of additional loci and phenotypes still await gene elucidation [2]. Current evidence supports the concept that the CCDDs are primarily due to neurogenic disturbances of brainstem or cranial nerve development [2].

The cranial sensory ganglia can be separated into two groups according to the origin of their constituent neurons. The proximal ganglia originate from the trigeminal and otic placodes and the neural crest, whereas the distal ganglia are derived from the other epibranchial placodes (facial, glossopharyngeal and vagus placode) [3]. Hence, the proximal ganglia include the ganglia for cranial nerves V, VIII and XI (trigeminal, vestibulo-cochlear and accessory nerves). The distal ganglia include the geniculate ganglion for cranial nerve VII, and cranial nerves IX and X are formed by both proximal (IXth and Xth, superior-jugular) and distal (IXth, petrosal and Xth, nodose) ganglia [4]. Cranial nerve V (trigeminal nerve) includes motoric nerve fibers in addition to sensory ones; these are derived from the nucleus motorius nervi trigemini in the rhombencephalon. Cranial nerve VI (abducens nerve) does not contain sensory nerve fibers. Neuronal development of all cranial sensory ganglia requires different basic helix-loop-helix transcription factors [5], including neurogenin1 (neurog1) and neurogenin2 (neurog2) [6]. Altered expression of neuronal differentiation helix-loop-helix transcription factor genes deranges neuronal differentiation in the central and the peripheral nervous system [5]. Studies in null mutant mice for *neurog1* and *neurog2* revealed a developmental halt of neurogenesis in cranial sensory ganglia at earliest stages [7]. Neurog1 was essential for the formation of the proximal cranial sensory neurons and neurog2 was found to be more involved in control of the distal cranial sensory neurons [8-10].

Clinical findings in CCDDs can include hearing loss and anatomic deviations of the cochlea and the vestibular apparatus [1]. Disturbances in embryogenesis of the otic placode can lead to sensorineural hearing loss and cochlear malformation. A common malformation of the cochlea is Mondini dysplasia, which is characterized by a cochlea of only 1.5 turns instead of the normal 2.5 turns. The interscalar septum between the middle and the apical coil is absent, leading to a common apical chamber with cystic dilatation. The vestibular system is affected in some cases [11]. Sensorineural hearing loss can also be caused by developmental disorders of the cranial sensory ganglia and the vestibulo-cochlear nerve.

Here we report on a 6-year-old boy with consanguineous parents displaying numerous clinical features, including profound sensorineural hearing loss, bilateral aplasia of cranial nerve VIII, cochlear hypoplasia (incomplete Mondini dysplasia), a severe disorder of oral motor function and torticollis. Genomic analysis identified a homozygous 5q31.1 deletion spanning 115 kb and including the *Neurogenin1* gene (*NEUROG1*). The congenital palsy of cranial nerves V (causing the severe gulp and mouth motor disorder) and VIII (causing the deafness) represents a congenital cranial dysinnervation disorder or Moebius syndrome variant and we suggest *NEUROG1* as the likely causative gene in this boy.

## Methods

This research was performed with the approval of the Ethics Board of the University Medical Center Mainz and in compliance with the Helsinki Declaration.

### Clinical report

**Figure 1:**
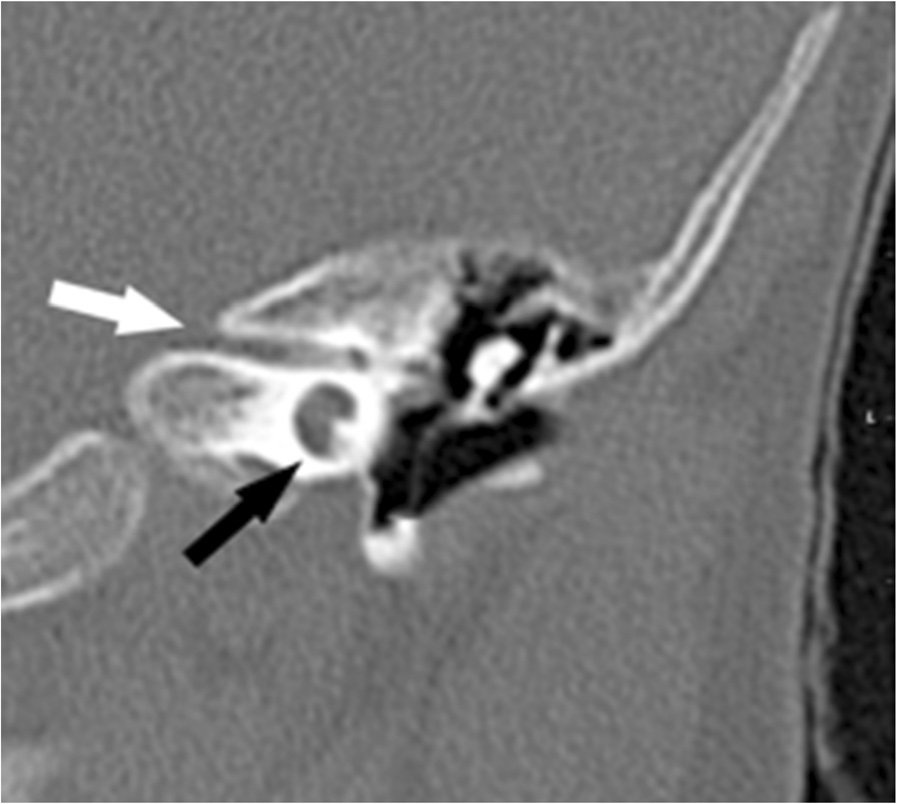
**High resolution CT of the left petrosus bone.** The coronal view demonstrates the widened single basal cochlear turn (black arrow) and the narrowing of the auditory canal (white arrow). The patient did not show an enlarged vestibular aqueduct (EVA) but similar findings of the right petrosus bone.

The 6-year-old proband was the first child of healthy consanguineous Turkish parents. His parents were second cousins. The parents reported that their common grandfather developed hearing impairment before age 50 years and that a great-granddaughter of their grandfather´s sister had received cochlea implants due to congenital deafness. Further data were not available. The proband was born by vacuum extraction after an uneventful pregnancy of 40 weeks gestation. The birth weight was 3,750 g (50th to 75th percentile), length 52 cm (50th percentile), head circumference 35 cm (50th percentile) and Apgar scores were 8 and 9 at 1 and 5 minutes. After birth, he was ventilated for one day and treated with antibiotics due to suspected neonatal sepsis. At age 4 months, he was hospitalized because of episodes of screaming followed by sudden tonus loss. Global developmental delay, muscular hypotonia and torticollis were noticed. A metabolic disease was suspected but no diagnosis was made. A brainstem evoked response audiometry (BERA) test at age 8 months indicated profound bilateral deafness. Cranial computed tomography imaging showed bilateral stenosis of the internal auditory canal with a maximum diameter of 1.1 mm and a hypoplastic cochlea with only a single widened cochlear turn (Figure 1). Magnetic resonance imaging confirmed the abnormalities and Mondini malformation was diagnosed. The vestibulocochlear nerve was assumed to be aplastic because no nerval structure reaching the cochlea could be identified. At age 8 months the boy was fitted with hearing aids and at age 12 months with a cochlear implant (CI) on the left, but without any improvement in hearing or speech acquisition. At age 2 ½ years he began to walk freely. At age 4 years, he was fitted with a cochlear implant on the right, but again without improving hearing or speech.

**Figure 2:**
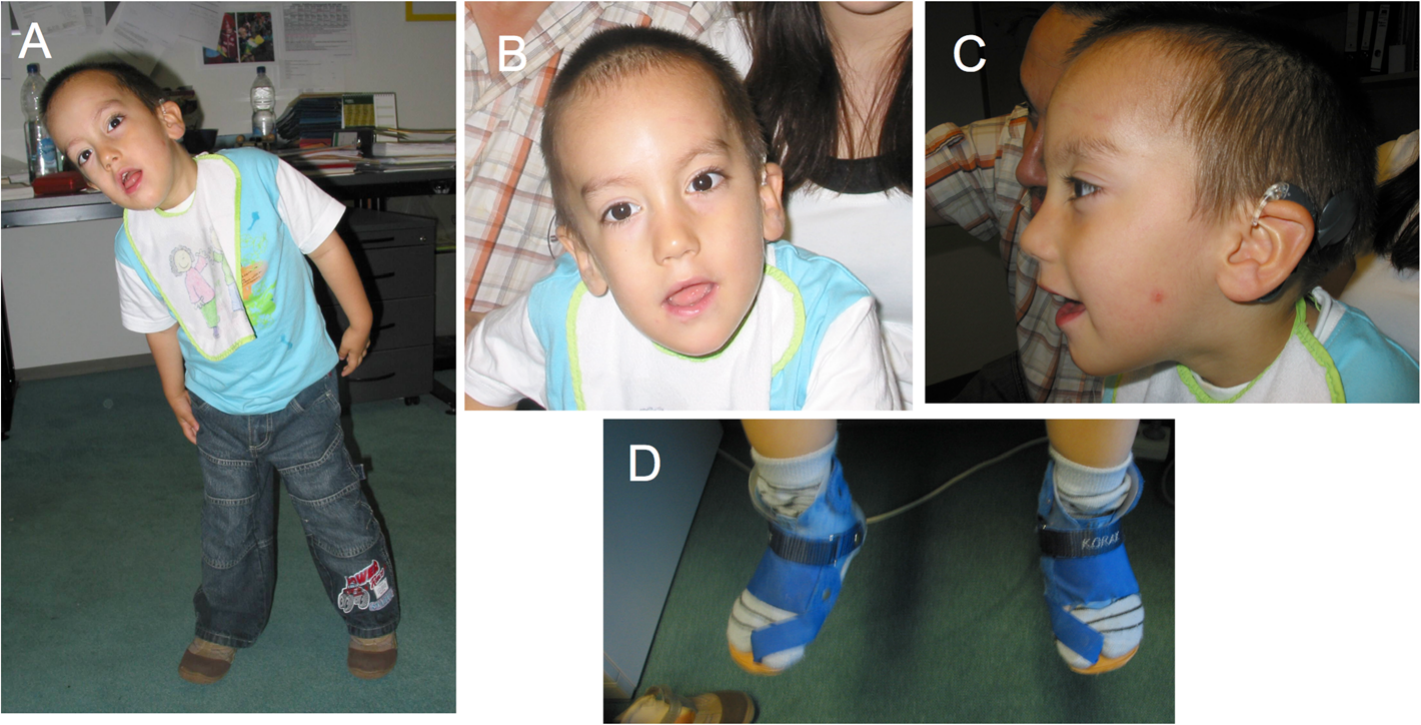
**The proband at age 3 ½ years.** Note (**A**) hypotonic appearance, balance disorder and torticollis, (**B**, **C**) scaphocephaly, high arched eyebrows, elongated palpebral fissures and low-set posteriorly rotated ears, and (**D**) orthopedic aids for correction of faulty foot posture.

When last seen at age 5 11/12 years, his length was 110 cm (5th percentile), weight was 21 kg (50th percentile) and head circumference 53 cm (75th percentile). He had plagioscaphocephaly, low-set posteriorly rotated ears, high arched eyebrows, elongated palpebral fissures, high and very narrow palate, funnel chest, sacral dimple, and hypoplastic genitalia. He wore ortheses because of faulty foot posture. His mother reported that he could ride a tricycle, but not a bicycle. He could stand on one leg only for a few seconds and an equilibrium disorder was assumed (Figure 2). Neurological findings included muscular hypotonia, increased salivation and a severe gulp and mouth motor disorder. He could not chew and needed mashed food. Intelligence testing showed large variations in results: his IQ was 70 using the SON-R (Snijders-Oomen non-verbal intelligence test) but 92 using the CPM-Raven (Raven´s Coloured Progressive Matrices), indicating normal abilities to think clearly and make sense of complexity (eductive ability), plus normal abilities to store and reproduce information (reproductive ability).

At age 1 year, karyotyping and mutation analysis of the *GJB2* and *GJB6* genes were performed with normal results. At age 3 years, a subtelomere screen using multiplex ligation-dependent probe amplification (MLPA) yielded normal results. Genome-wide microarray and NGS analyses were performed on samples taken at age 5 11/12 years.

### Genome-wide microarray analysis

The proband was among >200 patients studied by us using genome-wide microarray analysis. The Affymetrix Genome-Wide Human SNP Array 6.0 (~1,850,000 probes, 1.7 kb average distance between neighbouring probes) was used with the GeneChip Genome-Wide SNP Assay Kit 6.0 (Affymetrix, Santa Clara, CA). Data were analysed with the Genotyping Console 4.0 software (Affymetrix). Detection of CNVs required at least 5 consecutive abnormal probes spanning at least 100 kb in length. Results were verified using quantitative real-time PCR (qPCR).

### Mapping of the precise molecular breakpoints using next generation sequencing

**Figure 3:**
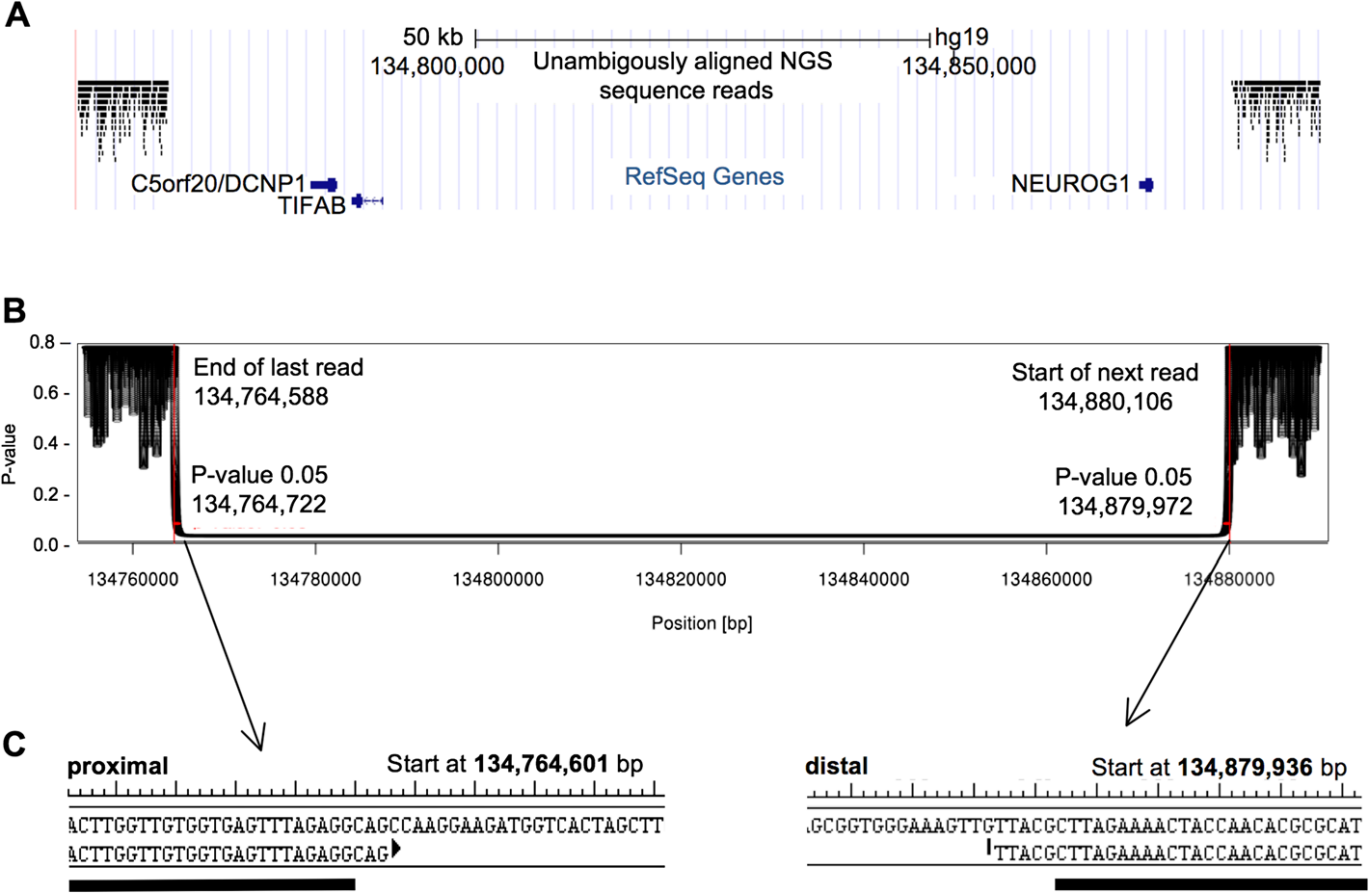
**Definition of the homozygous deletion by NGS and breakpoint-spanning PCR analysis.** (**A**) The locus, showing aligned reads (black bars) and RefSeq genes (blue). (**B**) A plot showing a p-value representing the likelihood that a genomic position is not in a homozygous deletion. (**C**) The sequence at the start and stop of the homozygous deletion; the black bars represent the location of primer sequences.

Using one flow cell lane of an Illumina HiSeq 2000, we generated 115,420,519 50 nt single end reads, with 101,701,184 reads passing standard quality filters. Reads were aligned to the human reference genome (GRCh37/hg19) using the Bowtie algorithm (version 0.12.7) [12]. 7,417,427 (7%) of the reads failed to align and 17,125,015 (17%) aligned to multiple locations and were removed, leaving 77,455,596 (76%) unambiguously aligned reads. After sorting reads based on genomic position using the samtools software package [13], breakpoints were determined using a statistical approach implemented in the R programming language. Based on the existing genetic and microarray data, we expected a homozygous deletion and thus expected no sequence reads to align to the deleted region. Away from the suspected deletion and outside of centromeres, we determined the distribution of a genomic position to the closest sequence read. Using this distribution, we determined the likelihood that a given position was in a homozygously deleted locus: a genomic position far from the closest sequencing read is more likely to be in a deleted locus. For a given position, the assigned p-value represents the fraction of all genomic positions that are an equal or greater distance from the closest read. The region identified by the Affymetrix SNP 6.0 GeneChip and the FREEC algorithm was examined in detail, including the aligned reads and the resultant p-values (Figure 3A, B) and identified a region where no reads aligned. 

The precise deletion sequence locus was determined using PCR with primers designed based on the NGS results, GCTTTTTCTCCTAAATTCTCTGG (forward) and TCAGCCATCTTTGTTGTTTCC (reverse) (Figure 3C). PCR was performed according to standard protocols. The reaction mixture consisted of 2.5 μl 10x PCR buffer, 2.5 μl 50 mM MgCl2, 2.5 μl 10 mM dNTP mix, 1.0 μl (100 ng) of each forward and reverse primer, 0.5 μl (2.5 U) Taq polymerase, 14 μl PCR-grade water und 100 ng template DNA. PCR amplification was carried out with an initial denaturation step at 94°C for 3 min, 35 cycles of 94°C for 30 sec, primer-specific annealing temperature of 55°C for 30 sec, 72°C for 60 sec and a final extension step at 72°C for 10 min. Following Exo/SAP digestion, PCR products were sequenced using the CEQ DTCS Quick Start Kit (Beckman Coulter, Krefeld, Germany) and separated on a Beckman Coulter CEQ 8000 Genetic Analysis System. Sequencing data were analysed using the BLAST program (http://www.ensembl.org/Homo_sapiens/blastview and http://www.ncbi.nlm.nih.gov/BLAST).

## Results

### Results of microarray analysis

**Figure 4:**
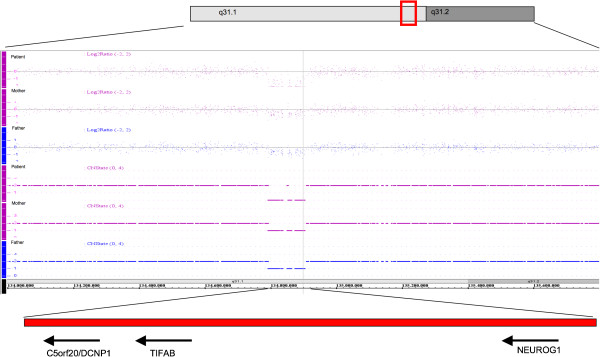
**Results of microarray analysis in the patient.** The homozygous deletion of chromosome 5q31.1 spans from 110.787 kb to 116.796 kb and includes three genes, *C5ORF20* (*DCNP1*)*, TIFAB* and *NEUROG1*. Both parents carried the same chromosomal microdeletion in heterozygous state.

Microarray analysis showed a homozygous deletion arr 5q31.1(134,764,990–134,875,777)x0 (GRCh37/hg19, ISCN2009) in the patient and identical but heterozygous deletions in the parents (Figure 4). The deletions were confirmed using qPCR. The proximal breakpoint on chromosome 5q31.1 was identified between 134,764,553 and 134,764,990 bp and the distal breakpoint between 134,875,777 and 134,881,349 bp (GRCh37/hg19), indicating a deletion size between 110,787 bp (minimum size) and 116,796 bp (maximum size) by microarray analysis (6,009 bp uncertainty).

### Results of the molecular breakpoint mapping using NGS and PCR analysis

Based on the NGS data for chromosome 5 (Figure 3), the proximal and distal breakpoint on 5q31 mapped between 134,764,588 and 134,764,722 bp (GRCh37/hg19) and 134,879,972 and 134,880,106 bp (p ≤ 0.05), respectively, indicating a deletion sized between 114,922 bp and 115,518 bp (269 bp uncertainty). By use of a breakpoint spanning PCR, we then mapped the precise breakpoints to 134,764,601 bp (last preserved base, proximal breakpoint) and 134,879,936 bp (first preserved base, distal breakpoint), indicating a deletion size of (exactly) 115,334 kb. The finding matched well with the approximate deletion interval established by microarray analysis and confirmed that the deletion interval comprised three genes, *NEUROG1*, *TIFAB* and *DCNP*1, encompassing all exons and likely the corresponding promotor regions (Figure 4).

## Discussion

We described a boy with cochlear hypoplasia, multiple neurogenetic abnormalities (profound sensorineural hearing loss, balance disorder, disorder of oral motor function, torticollis, and mild developmental delay) and a homozygous deletion of chromosome 5q31.1 spanning 115,334 kb. The deletion predicted a nullisomy (homozygous loss) for the *Neurogenin1* gene (*NEUROG1,* also called *Neurogenic differentiation factor 3*) and two other genes, *DCNP1* and *TIFAB*. To our knowledge, there has been no previous report of a homozygous deletion including these genes. In humans, none of these genes (*NEUROG1*, *DCNP1*, *TIFAB*) was previously associated with the clinical signs observed in this patient, but findings in Xenopus and mice make *NEUROG1* an excellent candidate gene [14].

The *neurog1* and *neurog2* genes and corresponding proteins have been well studied in vertebrates. In Xenopus, *neurog1* was shown to be essential for the expression of a cascade of downstream proteins that induce neuronal differentiation. Overexpression of *neurog1* induced a conversion of ectodermal cells into neurons during embryonic development [14]. In mice, expression of *neurog1* was found to be restricted to the nervous system. *Neurog1* showed high expression levels in developing sensory but not in autonomic ganglia [14]. In the central nervous system, *neurog1* and *neurog2* displayed overlapping expression patterns and functional redundancy in various regions of the brain [15]. In the peripheral nervous system, *neurog1* and *neurog2* displayed specific properties. *Neurog1* and *neurog2* were required during different phases of neurogenesis and for the development of different classes of sensory neurons [9]. The neurog1 protein was found to be essential for the development of proximal sensory ganglia and for neurons forming from the trigeminal and otic placodes [8]. These and other observations in knock-out mice are in excellent agreement with our clinical and radiological findings in the patient, which included a truncation or severe hypoplasia of the vestibulo-cochlear (VIIIth cranial) nerve. In the *neurog1−/−* mouse embryos, similar malformations of peripheral neural structures with absence of the vestibular-cochlear ganglion and of all afferent, efferent, and autonomic nerve fibers of the VIIIth cranial nerve were reported [10]. The efferent and autonomic fibers were thought to be lost as a secondary effect due to the absence of the afferents [10].

Furthermore, the anatomical deviations in the inner ear of the patient correspond to those in the knockout mice. The boy’s internal auditory canal was narrowed and the cochlea was hypoplastic with only one single widened cochlear turn. In the *neurog1−/−* mutant mice, the inner ear showed an overall reduction in size and the cochlea only had 1.25 turns, as opposed to 1.75 turns in the control littermates [10]. Moreover, the patient presented with a balance disorder, and the vestibulo-cochlear system of the *neurog1−/−* mutant mice showed a distinct missing utriculosaccular duct with only a small saccular recess [10].

This patient and the *neurog1−/−* knockout mice also shared the severe disorder of oral motor function. The boy was unable to swallow and to chew food and showed increased salivation and speech difficulties, while all newborn *neurog1−/−* mice were unable to suckle, lacked milk in their stomachs and died within 12 hours after birth [10]. Ma et al. suggested that the absence of the trigeminal ganglia and associated defects of the Vth nerve resulted in a lack of sensory innervation and neonatal lethality by interfering with suckling. Correspondingly, we assume a malfunction of the Vth cranial nerve in the boy that could be caused by lack of sensory innervation or a missing motor innervation due to defective development of the rhombencephalon, which is the origin of the motoric fibers of the Vth cranial nerve. Taken together, the anatomic and functional abnormalities in mice perfectly match the symptoms in this patient. Therefore, we strongly infer that *Neurog1* is a neuronal determination gene for the cranial sensory neurons that give rise to cranial nerves V and VIII in numerous vertebrates including Xenopus, mouse and humans.

In contrast, we consider *TIFAB* and *DCNP1* unlikely candidate genes for the abnormalities in this patient. *TIFAB* is highly expressed in spleen and inhibits *TIFA*-mediated cellular functions by inducing a conformational change in the TIFA protein [16]. TIFAB impedes activation of NF-kappaB and acts as a negative regulator of TRAF6-induced cellular functions such as B cell proliferation and maturation of macrophages [17]. *DCNP1* is a genetic factor that has been reported to be involved in asthma susceptibility. A case–control study among subjects with asthma revealed an association of variants of *DCNP1* with serum immunoglobulin E (IgE) levels [18]. *DCNP1* may also play a role in the pathogenesis of depressive disorders because it was shown to enhance corticotropin-releasing hormone expression in the hypothalamic paraventricular nucleus. Hence, *DNCP1* interferes with hypothalamic stress response and may contribute to the pathogenesis of major depression [19].

The clinical findings in our proband perfectly match with those in patients with CCDD, especially of the HOXA1 spectrum: among other symptoms, these patients show severe bilateral sensory-neural hearing loss due to absence of the cochlear and vestibular apparatus, often accompanied by absence of the eighth cranial nerve [1]. Every causative gene characterized in context of CCDDs is associated with neuronal development at the nuclear, brainstem, or peripheral nerve level [1,2]. For example, the responsible gene in Duane Retraction Syndrome (*CHN1*) is involved in ocular motor axon path finding of the sixth nerve. Homozygous mutations in *HOXA1* cause an early and profound brainstem patterning defect and heterozygous mutations in *KIF21A*, which are causative for congenital fibrosis of the extraocular muscles type 1, lead to disturbance of anterograde organelle transport in neuronal cells [2].

Taken together, the clinical and molecular genetic findings in our proband most closely match the term congenital cranial dysinnervation disorder and there are also analogies to Moebius syndrome and its variants (MIM 157900). In a large study of Moebius syndrome including 37 Dutch patients, the following clinical observations are consistent with those observed in our proband: feeding problems at birth due to insufficient suckling or swallowing, nasal dysarthria and delayed language development, congenital deafness, motor disabilities, and malformations of the extremities of variable severity. A lack of sensation of the lips, cheek, forehead and cornea in some patients indicated a defect of the sensory root of the trigeminal nerve [20]. Other authors have described external ear deformities, deafness and pharyngeal involvement in patients with Moebius syndrome variants [21-23].

Moreover, the clinical observations made in our proband partially correspond with those described in patients harboring the homozygous *HOXB1* c.619C > T mutation [24]: the phenotype included bilateral facial palsy, hearing loss, and strabismus. Two affected brothers from consanguineous parents showed a “masked facies” without any facial movement, sensorineural hearing loss, feeding difficulties and speech delay. MR imaging of the older brother revealed bilateral absence of the facial nerve and bilateral cochlear malformation with abnormal tapering of the basal turn. In contrast to our case, the vestibulocochlear nerve was preserved on both sides. An affected brother and sister from another family not known to be consanguineous also presented with bilateral facial weakness and sensorineural hearing loss [24].

The human NEUROG1 maps within the interval of the DFNB60 locus for non-syndromic autosomal recessive hearing loss on 5q22-q31, but linkage data have excluded *NEUROG1* from being causative in the DFNB60 patients (Michael S. Hildebrand and Richard J.H. Smith, Department of Otolaryngology - Head and Neck Surgery, University of Iowa, Iowa City, IA, USA, personal communication). However, given its large size (35 Mb, >100 genes), the 5q22-q31 area could well harbor more than one deafness gene.

## Conclusions

This is the first report on phenotypes associated with homozygous mutations or deletions of the human *NEUROG1* gene. We find, the clinical findings in this patient are in excellent coincidence with findings in frog (Xenopus) and mouse embryos lacking neurog1. The patient`s parents were phenotypically normal and carried the deletion in a heterozygous state, indicating autosomal recessive inheritance. We propose that congenital cranial dysinnervation disorders involving cranial nerves V and VIII may be caused by the presence of two null alleles for the human *NEUROG1* gene in a subset of patients. Further evaluation of the frequency of *NEUROG1* mutations in patients with congenital cranial dysinnervation disorders is needed before clinical recommendations can be given.

## Competing interests

The authors declare that they have no competing interests.

## Authors’ contributions

JCS carried out sequencing and drafted the manuscript. AKL and AK were the pedaudiologists in charge of the child and contributed the pedaudiologic data. DG performed the array analysis and helped with the figures. AP took part in writing the manuscript, it contains parts of her dissertation. JC, SD and UZ carried out the NGS analysis. WMF was the neuroradiologist in charge and contributed the corresponding clinical data. OB was the clinical geneticist of the child and family, he was the one who first noticed the resemblance to Moebius syndrome, initiated and coordinated molecular studies and literature research and helped to draft the manuscript. All authors read and approved the final manuscript.
